# 888. A Predictive Model for Subtherapeutic Vancomycin Troughs in Adults

**DOI:** 10.1093/ofid/ofac492.733

**Published:** 2022-12-15

**Authors:** Sarah Crisp, Nabin K Shrestha, Andrea Pallotta, Janet Wu, Christine Ahrens, Pooja Cerrato

**Affiliations:** Cleveland Clinic, Cleveland, Ohio; Cleveland Clinic, Cleveland, Ohio; Cleveland Clinic, Cleveland, Ohio; Cleveland Clinic, Cleveland, Ohio; Cleveland Clinic, Cleveland, Ohio; Cleveland Clinic, Cleveland, Ohio

## Abstract

**Background:**

Vancomycin is a first line agent in the treatment of methicillin-resistant *Staphylococcus aureus* infections. The ability to predict sub-therapeutic troughs would limit potential for development of resistance. The purpose of this study was to develop a predictive model for sub-therapeutic vancomycin troughs in adults.

**Methods:**

This retrospective cohort study included adults 18 years and older ordered a pharmacy to dose vancomycin consult. Exclusion criteria included patients with cystic fibrosis, pregnancy, no steady-state trough, or required dialysis during therapy. Patients with sub-therapeutic vancomycin troughs (< 10 mg/L) were compared to patients with therapeutic troughs (10-20 mg/L). A K-nearest neighbors (KNN) regression model to predict an initial sub-therapeutic vancomycin level based on age, gender, race, BMI, loading dose, frequency, creatinine clearance, and institutional vancomycin dosing guideline adherence was developed and validated using 10-fold cross validation. The association of vancomycin dosing guideline adherence with initial sub-therapeutic vancomycin trough was evaluated in a multivariable logistic regression model adjusted for age and creatinine clearance.

**Results:**

A total of 1,615 subjects were included; 494 (30.5%) subjects experienced a sub-therapeutic trough. A KNN regression model to predict an initial sub-therapeutic vancomycin level had an area under the ROC curve of 0.82, and an accuracy of 0.77 (95% CI 0.75-0.79, baseline accuracy 0.69). After adjusting for age and creatinine clearance, guideline dosing adherence was associated with significantly lower odds of an initial sub-therapeutic vancomycin level compared to guideline dosing non-adherence (OR 0.37, 95% CI 0.20-0.66, p-value 0.001).

**Figure:**
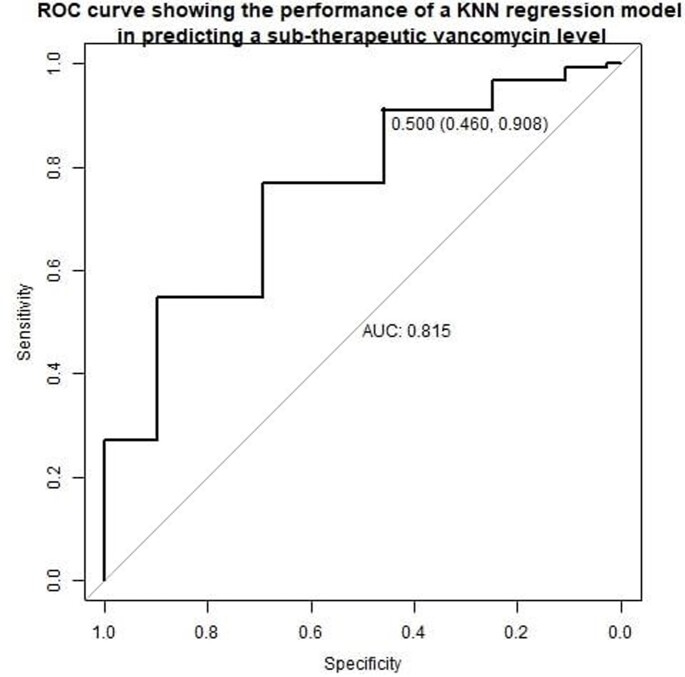

**Conclusion:**

A predictive model, based on readily available data points, for an initial sub-therapeutic vancomycin level was reasonably accurate. Adherence to the institutional vancomycin dosing guideline was associated with greater than 60% lower odds of an initial sub-therapeutic vancomycin level.

**Disclosures:**

**All Authors**: No reported disclosures.

